# Guillain–Barré Syndrome After Malaria: A Case Report of a 7‐Year‐Old Child With Asymmetric Onset of Acute Motor Axonal Neuropathy

**DOI:** 10.1002/ccr3.72592

**Published:** 2026-04-23

**Authors:** Muath Ibrahim Mohammed Abusaada, Mustafa Mohamed Ibrahim Ali, Muotaman Mohammed Abdalla Adam, Mohammed Hishameldeen Omer Ali, Mohamed Abdallah Mohamed Baraka, Amjad Ali Mohamedelhassan Bakheit, Khabab Abbasher Hussien Mohamed Ahmed, Isameldin Yousif Khidir Mohmed

**Affiliations:** ^1^ Faculty of Medicine and Health Sciences Nile Valley University Atbara Sudan; ^2^ Faculty of Medicine University of Khartoum Khartoum Sudan; ^3^ Faculty of Medicine University of Science and Technology Omdurman Sudan

**Keywords:** AMAN type, asymmetric weakness, Guillain–Barré syndrome, malaria, pediatrics, Sudan

## Abstract

This case highlights that acute motor axonal neuropathy can present with asymmetric, relapsing weakness in children. In malaria‐endemic and resource‐limited settings, clinicians should consider atypical Guillain–Barré variants early, as timely recognition and treatment can significantly improve outcomes despite diagnostic and systemic constraints.

## 1 Introduction

Guillain–Barré syndrome (GBS) is an immune‐mediated polyradiculoneuropathy affecting the peripheral nervous system, characterized by the sudden onset of progressive, typically symmetrical limb weakness, often accompanied by sensory symptoms [1]. Atypical presentations, including marked asymmetry or an unusual clinical course, may delay recognition and broaden the differential diagnosis.

GBS is most commonly triggered by preceding infections. Although *Campylobacter jejuni* remains the leading antecedent worldwide and has been reported to account for a substantial proportion of cases in Africa [2], dengue fever and malaria have also been reported as preceding infections and are particularly relevant in endemic countries such as Sudan [2, 3, 4, 5]. In such settings, where acute febrile illnesses are common, early recognition of neurological deficits occurring during or after febrile episodes is important to enable timely diagnosis and treatment.

Diagnosis is primarily clinical, supported by cerebrospinal fluid analysis and electrophysiological studies to confirm GBS and differentiate demyelinating from axonal subtypes [6]. Acute motor axonal neuropathy (AMAN) is a pure motor axonal variant in which antibody‐mediated injury targets motor axons and nodes of Ranvier, commonly associated with anti‐ganglioside antibodies (e.g., GM1, GD1a), without primary demyelination. Clinically, AMAN may progress rapidly and may demonstrate relatively preserved or brisk reflexes, while autonomic dysfunction is less prominent [7].

GBS is generally monophasic; however, relapses or treatment‐related fluctuations have been described in a minority of patients [8]. Pronounced asymmetry is atypical in AMAN and can further complicate diagnosis, particularly in pediatric patients and in post‐infectious contexts.

Relapsing AMAN with prominent asymmetry in children remains poorly described, particularly when occurring in temporal association with infections such as malaria or dengue in endemic areas. We report a 7‐year‐old boy with asymmetric, relapsing AMAN 3 weeks after a malaria infection, who initially improved after treatment but was readmitted 3 weeks later, highlighting the need to consider atypical post‐infectious GBS variants and to ensure close follow‐up after early clinical improvement.

## 2 Case History/Examination

A 7‐year‐old Sudanese male presented to the pediatric emergency department with progressive, asymmetrical, ascending weakness of the lower limbs. The right lower limb was affected 10 days ago, followed by the left lower limb 2 days later, and both upper limbs became involved 3 days ago. The patient's history revealed malaria 3 weeks ago and a cold with flu‐like symptoms 2 weeks prior, along with a longstanding history of proximal muscle weakness since age 4.

There was no history of consanguinity or family history of neuromuscular disorders. The proximal weakness had been non‐progressive according to the family, and developmental milestones were appropriate for age. There were no clinical features suggestive of muscular dystrophy such as calf hypertrophy or Gowers' sign. Baseline laboratory investigations, including muscle enzyme levels, were within normal limits. Although genetic testing was not performed due to resource limitations, the acute presentation and electrophysiological findings were consistent with superimposed AMAN rather than a primary chronic myopathic disorder.

His vaccination record is current, including polio immunization. Family, psycho‐social history, and genetic background showed no relevant associations.

His Physical examination was performed; the patient had normal vital signs. Limb examination revealed hypotonia, a power grade of 3 with distal weakness more than proximal, and areflexia, with these findings being more pronounced in the lower limbs than the upper limbs. Sensation and cranial nerve function were intact. The patient experienced no back pain, and there was no respiratory, sphincteric, or bulbar involvement.

On detailed neurological examination using the Medical Research Council (MRC) scale, motor power was assessed as follows: right lower limb—hip flexion 3/5, knee extension 3/5, ankle dorsiflexion 2/5; left lower limb—hip flexion 4/5, knee extension 4/5, ankle dorsiflexion 3/5; right upper limb—shoulder abduction 4/5, elbow flexion 4/5, wrist extension 3/5; left upper limb—shoulder abduction 4/5, elbow flexion 4/5, wrist extension 4/5. Deep tendon reflexes were absent in both lower limbs and reduced in both upper limbs. Plantar responses were flexor bilaterally. Sensory examination was intact to all modalities.

## 3 Differential Diagnosis, Investigations and Treatment

The patient was admitted after an initial assessment for suspected Guillain–Barré syndrome (GBS), as his history made the diagnosis of poliomyelitis less likely. Other differential diagnoses considered included transverse myelitis, acute flaccid myelitis, spinal cord compression, myasthenia gravis, metabolic neuropathies, and periodic paralysis. Transverse myelitis and spinal cord pathology were unlikely due to the absence of sensory level, sphincter involvement, back pain, or upper motor neuron signs. Acute flaccid myelitis was considered less likely given preserved cranial nerve function and CSF findings of albumin‐cytological dissociation. Myasthenia gravis was excluded based on the absence of fatigability, ocular or bulbar involvement, and the presence of areflexia. Baseline laboratory investigations were within normal limits, making metabolic causes unlikely. CBC showed Hb 11.9, WBC 390, lymphocytes 26.3, granulocytes 73.7, MCV 71.5, MCH 25.3, and platelets 414. CK was 92, within normal range. Consequently, a CSF analysis was performed, which showed albuminocytological dissociation (Table 1), followed by a nerve conduction study that indicated severe axonal motor polyneuropathy (Table 2), confirming the patient's condition as the AMAN variant of GBS based on Brighton Criteria for GBS and Electrophysiological Criteria for AMAN Subtype.

**TABLE 1:** CSF analysis showing albumin‐cytological dissociation (consistent with GBS).

CSF analysis			
Parameter	Result	Reference range	Notes/Interpretation
Volume/Appearance/Color	Fresh sample, clear, colorless	Clear/Colorless	Specific gravity Q.N.S
Glucose (mg/dL)	69	60–80	Within normal range
Protein (mg/dL)	67.5	15–40	Elevated; albumin‐cytological dissociation
WBC/Differential	0	0–5	Not reported
RBC	0	0	Not reported
Culture/Growth	No growth after 48 h at 37°C	—	Negative for bacterial growth

**TABLE 2:** Nerve conduction study results showing severe axonal motor polyneuropathy (consistent with AMAN variant of GBS).

Nerve conduction study results				
Type	Nerve	Amplitude	Distal latency (DL)	Conduction velocity (CV)
Motor nerve conduction	Left median	Markedly reduced	Normal	Normal
	Left ulnar & radial	Reduced	Normal	Normal
	Both peroneal	Markedly reduced	Normal	Normal
	Both tibial	Markedly reduced	Normal	Normal
Sensory nerve conduction	Left median	Normal	Normal	Normal
	Left ulnar & radial	Normal	Normal	Normal
	Right sural	Normal	Normal	Normal

The patient had to travel from his home state, covering a significant distance to reach our facility due to the ongoing war, which rendered the local facilities in his home state non‐functional, resulting in a delay in his presentation and the start of management.

After the diagnosis was confirmed, the patient received intravenous Immunoglobulin (IVIG) at a dose of 10 g, infused over 4 h for 4 days (a total of 40 g). Following treatment, the patient's condition improved markedly, with only mild weakness remaining. He was discharged with a referral to physiotherapy and scheduled for weekly follow‐up.

## 4 Conclusion and Results (Outcome and Follow‐Up)

During follow‐up 3 weeks post‐discharge, the patient exhibited right‐sided weakness with hypotonia and hyporeflexia on examination. Baseline investigations were normal. This deterioration following initial improvement was considered consistent with treatment‐related fluctuation (TRF), a recognized phenomenon in Guillain–Barré syndrome that may occur within the first 8 weeks after onset. Although repeat IVIG is not routinely recommended in standard guidelines following a favorable initial response, it may be considered in cases of documented clinical relapse. After multidisciplinary reassessment and exclusion of alternative causes, the decision was made to administer a second course of IVIG. The patient received another dose of IVIG (10 g infused over 4 h for 4 days). After the second treatment regimen, the patient's condition improved significantly, and they were scheduled for regular follow‐up. The entire timeline of the patient's condition is available in Table 3.

**TABLE 3:** The entire timeline of the patient's condition.

GBS clinical timeline		
Time point	Event	Clinical details
T = −3 weeks	Started treatment for a malaria infection	Possible trigger of the patient's condition
T = −2 weeks	Common cold with flu‐like symptoms	
T = −10 days	Right lower limb weakness starts and progresses	Asymmetrical ascending lower limb weakness, starting with right lower limb followed by left lower limb before affecting upper limbs
T = −8 day	Left lower limb weakness starts	
T = −3 days	Both hands weakness	
T = 0 (April, 2025)	Patient present to Pediatric ER+initial assessment+admission	GBS Diagnosis; CSF analysis: albumin cytological dissociation; NCS: AMAN variant of GBS
T = +1 day	Initiation of treatment	Patient was started on IVIG and closely monitored
T = +1 week	Patient improved and discharge	Physiotherapy program started+weekly follow up
T = +3 weeks	Pediatrics referred patient with right lower limb and upper weakness	Decision by consultant to start another IVIG treatment regimen
T = +8 weeks	Follow‐up and improvement	Child performs his daily functions normally

## 5 Discussion

Classically, GBS is triggered following a GI or respiratory tract infection [9, 10, 11]. In malaria‐endemic regions, Malaria should be considered as a trigger for GBS in the presence of a febrile illness [11].

Classic infectious triggers include infection with *Campylobacter jejuni* being the most common among others such as *Mycoplasma pneumoniae* [12]. Some studies have reported malaria preceding GBS while some went to suggest Malaria as a cause of GBS [10, 13]. GBS is usually diagnosed clinically with or without the help of lab tests [14].

This case describes a 7‐year‐old Sudanese male who presented with asymmetrical ascending weakness 3 weeks after a malaria infection and 2 weeks after a respiratory infection, ultimately diagnosed as Guillain–Barré Syndrome (GBS) following clinical criteria and relevant lab tests for the disorder raising the question of “what was the trigger?”

Differential diagnosis at presentation included poliomyelitis as the asymmetric pattern of weakness was confusing in the beginning. However, the child was vaccinated. The subacute onset and progress of symptoms made the diagnosis of more rapid conditions such as poliomyelitis less likely. In addition, lab tests were consistent with GBS as nerve conduction studies showed reduced velocities in motor nerves which shifted the focus away from other differentials such as myopathies.

NCS showed reduction only in motor nerves sparing all sensory nerves marking the diagnosis as the AMAN variant of GBS. CSF analysis showed normal glucose and elevated protein in the absence of microorganisms. WBCs and RBCs were not seen and thus fall within the normal range. These findings were interpreted as albumin‐cytological dissociation which further supported the diagnosis of GBS.

In a case report published in 2025 from Ethiopia, the authors reported the case of a 16 year old female who presented with altered mentation and severe headache [3]. She was diagnosed with Falciparum malaria. Four days after her initial complaint—three after admission—she developed weakness and numbness in her lower extremities. Afterwards, the weakness ascended to involve both upper limbs. The patient had no history of gastrointestinal or respiratory infections. Ultimately, the diagnosis of GBS due to malaria was considered [3]. In our case report, GBS followed the diagnosis of malaria as well. The differences being the time of weakness symptoms development post infection, the pattern of weakness and the absence of sensory symptoms in our case. It is also important to note the respiratory infection in our case as a potential cause. In the Ethiopian case, IVIG treatment was not possible and the patient eventually recovered without the treatment [3].

An older case report from Sri Lanka from 1992 described a 45‐year‐old male patient who presented with a 3‐day history of fever, chills and headache. A diagnosis of Falciparum malaria was made [15]. The patient was admitted and 2 days after admission, he developed numbness and weakness of both lower limbs. The patient was examined and was found to have hypotonia in all four limbs. He had decreased reflexes overall and absent reflexes in the ankles. Touch and pinprick were impaired in both lower and upper limbs. He was diagnosed with GBS following the malaria infection. The author describes the case at the time as the first case report of GBS following malaria [15]. This case is different from ours in the pattern of weakness, sensory involvement and treatment as IVIG was not administered to the 45‐year‐old patient [15].

Considering valuable information obtained through the patient's history, his longstanding proximal muscle weakness was ruled out from causing his hospital presentation of this study. History had revealed that the proximal muscle weakness had plateaued long before the time of this case report. The absence of family history of a similar condition combined with the patient's physical exam, the presence of both malaria and a respiratory infection 2 and 3 weeks before the onset of weakness respectively, clinical criteria for GBS diagnosis (…) and lab tests such as CSF analysis and NCS were all considered and hence a ruling was made that it was unlikely that the patient's acute presentation was related to the longstanding history of proximal muscle weakness.

A case report from India mentioned their patient, a 65‐year‐old female, presented with sudden symmetrical lower limb weakness 1 week following high grade fever, chills and rigors. The weakness ascended to include the upper limbs. There was no preceding GI or respiratory tract infection. Plasmodium Vivax was detected in her blood and NCS showed prolongation of latencies in motor nerves. Diagnosis of GBS was established on the basis of clinical evaluation and lab testing. In our case, NCS showed normal distal latencies in motor nerves but showed marked reduction in amplitude. Another difference is that our patient developed weakness 3 weeks after malaria and 2 weeks after the respiratory infection. Similar to our patient, the patient from the Indian study had no sensory symptoms, no sphincteric or bulbar involvement [13].

One paper from Sudan published in 2002 studied the development of GBS following malaria infection. The paper observed 10 patients with GBS who were diagnosed with malaria prior to the development of neurological symptoms. The authors reported patients developing polyneuropathy between three and 30 days after manifesting with fever [16]. Our patient presented with weakness 3 weeks following the development of malaria, which falls within the timeframe observed by the 2002 study.

## Author Contributions

**Muath Ibrahim Mohammed Abusaada:** conceptualization, data curation, formal analysis, funding acquisition, investigation, methodology, project administration, resources, software, supervision, validation, visualization, writing – original draft, writing – review and editing. **Mustafa Mohamed Ibrahim Ali:** conceptualization, data curation, formal analysis, funding acquisition, validation, visualization, writing – original draft, writing – review and editing. **Muotaman Mohammed Abdalla Adam:** conceptualization, validation, visualization, writing – original draft, writing – review and editing. **Mohammed Hishameldeen Omer Ali:** conceptualization, validation, visualization, writing – original draft, writing – review and editing. **Mohamed Abdallah Mohamed Baraka:** conceptualization, validation, visualization, writing – original draft, writing – review and editing. **Amjad Ali Mohamedelhassan Bakheit:** conceptualization, validation, visualization, writing – original draft, writing – review and editing. **Khabab Abbasher Hussien Mohamed Ahmed:** conceptualization, data curation, validation, visualization, writing – original draft, writing – review and editing. **Isameldin Yousif Khidir Mohmed:** conceptualization, validation, visualization, writing – original draft, writing – review and editing.

## Funding

The authors have nothing to report.

## Ethics Statement

Ethical approval was not needed for this case report (as per our institute policies and regulations).

## Consent

Written informed consent was obtained from the patient's guardian to publish this report in accordance with the journal's patient consent policy.

## Conflicts of Interest

The authors declare no conflicts of interest.

## Data Availability

All the data that support the findings of this study are available from the corresponding author upon reasonable request.
